# Is there a role for carvedilol in the management of pediatric heart failure? A meta analysis and e-mail survey of expert opinion

**DOI:** 10.4103/0974-2069.52816

**Published:** 2009

**Authors:** Balu Vaidyanathan

**Affiliations:** Department of Pediatric Cardiology, Amrita Institute of Medical Sciences, AIMS, Ponekkara P.O., Kochi, Kerala - 682 041, India

## INTRODUCTION

Heart failure is a complex disorder characterized by widespread activation of various neurohormonal pathways resulting in increased circulating levels of catacholamines. In the short run, these circulating catacholamines may augment heart rate, blood pressure and myocardial contractility. However, chronic adrenergic stimulation leads to worsening of heart failure by inducing myocardial apoptosis and fibrosis.[1] Circulating catecholamines also cause peripheral vasoconstriction along with retention of salt and water, both of which are counterproductive. In addition, adrenergic stimulation may predispose to ventricular tachyarrhythmia and sudden cardiac death.

## BETA-BLOCKERS IN THE TREATMENT OF HEART FAILURE

Beta-blockers were first used in the management of congestive heart failure in adults more than 30 years ago.[2] Despite their beneficial effects in the study, beta-blockers remained contraindicated in patients with heart failure because of the side effects of bradycardia, hypotension and depression of myocardial contractility. However, in the last 20 years, a large number of randomized clinical trials proved without doubt the value of beta-blockers in improving outcomes in adult patients with chronic heart failure.[3–6] These trials have shown that beta-blocker therapy improves systolic function of the heart, leads to remodeling of the dilated ventricles, improves symptoms and reduces the risk of hospitalization and death. Recent guidelines for management of heart failure in adults recommend that beta-blocker therapy should be an integral part of the standard heart failure treatment regimens.[7] Several beta-blockers, including carvedilol, metoprolol and bisoprolol, were used in these trials. After the results of the COMET trial, carvedilol has become the beta-blocker of choice in adults with chronic heart failure.[8]

Carvedilol is a third-generation beta-blocker, which has an alpha-blocking action as well. Thus, in addition to the beta-blocking action (non-selective), it causes peripheral vasodilatation by the alpha-receptor blockade.[9] Carvedilol may have other potential beneficial effects in heart failure as an anti-oxidant and as an anti-proliferative agent.[9] It was the first agent in this class of drugs to be approved by the Food and Drug Administration in 1995 for the treatment of heart failure. At present, carvedilol is approved for use only in adult patients. Several small studies and case reports have documented its efficacy and safety in infants and children.[10–19] These studies eventually stimulated a large randomized control trial of carvedilol in pediatric heart failure by Shaddy *et al*. in 2007.[20] This drug review and expert opinion survey tries to summarize the current role of carvedilol in the management of pediatric heart failure in the light of the data from the recent clinical trials.

## PHARMACOKINETICS OF CARVEDILOL IN CHILDREN

The bioavailability after oral administration is 25-35% because of significant first-pass metabolism in the liver.[21] It is metabolized through aromatic ring oxidation and glucoronidation in the liver. Studies have shown that the elimination half-life is significantly shorter in children compared with adults (2.9 vs. 5.2 h), resulting in higher peak serum levels in children.[21] Because of rapid clearance, the trough levels may be lower in children as compared with adults.[20]

## ADVERSE EFFECTS AND DRUG INTERACTIONS

As with other beta-blockers, carvedilol is not recommended for patients with asthma or bronchospastic disease, diabetes, advanced heart block, sick sinus syndrome, acute heart failure and cardiogenic shock. Dose reduction is required in liver failure.[21] The most common side effects observed in pediatric studies include dizziness (19% of patients), hypotension (14%), headache (14%), vomiting (9%), fatigue or dyspnea (7%), edema (5%), chest pain, acid reflux, atrial flutter and syncope (in 2% each) (10). Worsening heart failure was observed in 4-11% of the cases.[11,20]

Drug interactions can occur with digoxin, cyclosporine and hepatic enzyme inducers like rifampicin. Concomitant use of digoxin and carvedilol reduces the digoxin clearance from the body and hence it is recommended that the digoxin dose be reduced by 25% when coadministered with carvedilol.[22]

## DOSING AND ADMINISTRATION

Based on the studies performed to date, carvedilol should be initiated in infants and children at a dose of 0.05-0.2 mg/kg/day, given in two divided doses.[21] The dose may be titrated at 1-2-week intervals to a target maintenance dose of 0.2-1 mg/kg/day, with a suggested maximum dose of 2 mg/kg/day. Parents should be instructed not to discontinue the drug abruptly and watch for dizziness on standing, especially during the first few weeks after starting the therapy.

The drug is available as 3.125, 6.25, 12.5 and 25 mg tablets. While there are no commercially available oral liquid formulations of carvedilol, a formulation can be prepared from the tablets. The tablets should be crushed and mixed with water and these formulations may be stored at room temperature for up to 12 weeks.[21]

## CLINICAL TRIALS OF CARVEDILOL IN CHILDREN

Unlike adult patients, very few randomized controlled trials have been performed using f beta-blockers in pediatric patients with heart failure. These studies have analyzed the effect of the addition of beta-blockers (mostly carvedilol) in patients already receiving standard heart failure treatment (digoxin, diuretics, ACE inhibitors).[10–19] Most of these studies included a small number of patients studied over a relatively short period of time (around 12 months) and used end points like improvement in the symptom class, left ventricular function (ejection fraction, fractional shortening and left ventricular end-diastolic and end-systolic dimensions and volumes) and plasma levels of circulating catecholamines and B-type natriuretic peptide (BNP).

Most of these studies reported a favorable impact of carvedilol on symptom class and left ventricular functional parameters (ejection fraction, fractional shortening and end-diastolic and end-systolic dimensions and volumes). Some studies reported survival benefit and reduced need for transplantation.[14,15] Giardini *et al*. reported significantly lower plasma concentrations of norepineprine, dopamine and aldosterone after 12 months of therapy with carvedilol.[18] Another study reported a significant increase in the cardiac uptake of iodine-123 metaiodobenzylguanidine, representing a significant improvement in cardiac adrenergic neuronal function with carvedilol treatment.[19] These initial studies concluded that carvedilol is a useful adjunct to standard therapy in children with heart failure. However, the International Society for Heart and Lung Transplantation practice guidelines for management of heart failure in children did not make a recommendation for the use of beta-blockers in patients with left ventricular dysfunction, giving the limited data available.[23]

## RANDOMIZED CONTROLLED TRIAL OF CARVEDILOL IN PEDIATRIC HEART FAILURE

The first and only prospective randomized control on use of carvedilol in children with heart failure was reported by Shaddy *et al*. in 2007.[20] This multi-centric randomized double blind, placebo control trial included 161 children and adolescents with symptomatic systolic heart failure from 26 US centers. In addition to conventional heart failure medications, patients were assigned into three groups – placebo, low-dose carvedilol group (0.2 mg/kg/dose BD if weight <62.5 kg or 12.5 mg BD if weight >65 kg) and high-dose carvedilol group (0.4 mg/kg/dose BD if weight <62.5 kg or 25 mg BD if weight >65 kg). Patients were stratified according to age, sex and ventricular morphology (systemic left or right ventricle or single ventricle); those with systemic left ventricle required an ejection fraction of less than 40% as an inclusion criterion. The primary outcome was a composite measure of heart failure outcomes; secondary outcome variables included individual components of the composite end point, echocardiographic measures and plasma BNP levels. The mean duration of treatment was 8 months.

The following were the major observations made from this study:

- No significant difference in the composite end point between the treatment groups based on percentage of patients who improved, worsened or was unchanged.
- There was a significant interaction between study drug and ventricular morphology, suggesting a beneficial trend for patients with systemic left ventricle versus a non-beneficial trend for those with systemic right or single ventricle.
- A significant proportion of younger patients (< 24 months) improved compared with those older than 24 months.
- The hazard ratios for mortality and hospitalization rates showed a trend favoring carvedilol over placebo, although the difference was not statistically significant.
- Echocardiographic parameters showed a statistically significant improvement in patients with systemic left ventricle, especially in the high-dose carvedilol group.
- The drug was generally well tolerated in most patients; worsening heart failure leading to drug withdrawal occurred in about 11% of the patients in the carvedilol group.
- The average trough concentrations of the drug were lower than that observed in adult studies, indicating a more rapid clearance of carvedilol in children.

This is the first major trial on beta-blockers in heart failure, which reported a lack of significant benefit on the composite end points studied. The authors postulate that inherent heterogenecity in the etiology of pediatric heart failure, different ventricular morphologies in the study patients and the possible high rate of spontaneous improvement in younger patients as possible reasons to explain the relative lack of benefit of carvedilol in their study. Moreover, the pharmacokinetics of carvedilol is different in children, resulting in more rapid drug clearance and lower trough serum levels of the drug in children, as was observed in this study.

The e-mail survey of expert opinion tried to address some of the issues that emerged after the publication of the study by Shaddy *et al*.[20] As in the previous surveys, a pre-designed questionnaire addressing some of the common issues regarding the use of carvedilol in pediatric heart failure was circulated to a panel of experts and their opinions were solicited. The Pediatric Cardiac Society of India had organized a consensus meeting on the management of congenital heart disease (including heart failure) in India at AIIMS, New Delhi on 13 September 2008.[24] The summary of the expert opinion through the e-mail survey and the consensus meeting are presented below.

### Do you recommend the use of carvedilol in the management of heart failure in children? If so, what would be the indications?

Most of the respondents (except one) felt that there is a definite role for carvedilol in the management of pediatric heart failure. Heart failure due to myocardial failure (dilated cardiomyopathy/myocarditis) was agreed upon as the only indication for use of carvedilol (level of evidence A). Although there are some studies reporting a favorable effect of carvedilol in heart failure due to left-right shunts,[25] all the experts who participated in the e-mail survey and the consensus meeting were not in favor of the use of beta-blockers in patients with left-right shunts or valve regurgitations.

### At what stage of treatment do you start carvedilol? Do you start along with diuretics, ACE inhibitors and digoxin or do you prescribe it sequentially?

The general consensus among all the respondents and experts who participated in the consensus meeting was to start the standard therapy of digoxin, diuretics and ACE inhibitors first and then initiate carvedilol once the maximum tolerated doses of the other drugs (especially ACE inhibitors) was achieved.

### What severity of heart failure would you consider as a contraindication for carvedilol treatment?

Most experts were reluctant to initiate carvedilol treatment in children who had advanced heart failure (NYHA class IV, fractional shortening <10%, requiring inotropic support).

### Do you prescribe carvedilol in patients with heart failure and systemic right ventricle or single ventricle?

Most of the experts who participated in the e-mail survey felt that carvedilol may not be useful in patients with systemic right ventricle (cTGA/post-atrial switch operation) or single ventricle. In the national consensus meeting, however, the use of carvedilol in patients with systemic right ventricle was considered as optional (level of evidence IIa).

### What is the dosing schedule? How do you titrate dose and how do you monitor therapy?

All the respondents were in agreement with the standard guidelines for starting carvedilol therapy in a low dose and then titrating the dose once a week toward the maximum tolerated dose. Most experts preferred to start the treatment in the hospital, although the dose titration maybe performed in the clinic setting. Standard methods (symptoms, drug tolerance, echocardiographic parameters) should be used to monitor response to treatment.

### What is your personal experience regarding the efficacy of carvedilol in pediatric heart failure with respect to symptoms, frequency of hospitalizations, ventricular function and mortality?

Most of the experts felt that carvedilol is useful in improving symptoms in children with heart failure. Some have observed improvement in ventricular function. However, the respondents were unclear about the more robust end points like mortality and frequency of hospitalizations.

### How frequently have you encountered worsening of symptoms of heart failure after starting carvedilol? What is your protocol for managing such patients?

Most experts felt that carvedilol is safe and often well tolerated in children with heart failure. One of the experts (Dr. S. S. Kothari) observed that worsening of heart failure may not be that infrequent after initiating carvedilol (up to 20% patients).

The protocol suggested for managing patients with worsening of heart failure is to withdraw the drug immediately and then use inotropes like milrinone or dopamine in the intensive care unit or oral enoximone in the ward (Dr. John Simpson). Reinitiation of carvedilol has to be performed very cautiously, at a lower dose with a slower titration.

## CONCLUSIONS

Carvedilol is a useful adjunct in the pharmacotherapy of heart failure in children, especially due to myocardial disease. In children with systemic left ventricle, there is clear evidence that carvedilol therapy improves symptoms and results in improvement in echocardiographic indices of ventricular function. In patients with systemic right ventricle or single ventricle, current data do not support the use of carvedilol for managing heart failure. Long-term prospective randomized controlled studies should address the impact of carvedilol treatment on more robust end points like mortality.
